# Cinematic Operation of the Cerebral Cortex Interpreted via Critical Transitions in Self-Organized Dynamic Systems

**DOI:** 10.3389/fnsys.2017.00010

**Published:** 2017-03-14

**Authors:** Robert Kozma, Walter J. Freeman

**Affiliations:** ^1^College of Information and Computer Sciences, University of MassachusettsAmherst, MA, USA; ^2^Department of Mathematical Sciences, University of MemphisMemphis, TN, USA; ^3^Department of Molecular and Cell Biology, University of California at BerkeleyBerkeley, CA, USA

**Keywords:** cinematic theory of cognition, AM pattern, criticality, phase transition, Freeman K set, Hebbian assembly, graph theory, neuropercolation

## Abstract

Measurements of local field potentials over the cortical surface and the scalp of animals and human subjects reveal intermittent bursts of beta and gamma oscillations. During the bursts, narrow-band metastable amplitude modulation (AM) patters emerge for a fraction of a second and ultimately dissolve to the broad-band random background activity. The burst process depends on previously learnt conditioned stimuli (CS), thus different AM patterns may emerge in response to different CS. This observation leads to our cinematic theory of cognition when perception happens in discrete steps manifested in the sequence of AM patterns. Our article summarizes findings in the past decades on experimental evidence of cinematic theory of cognition and relevant mathematical models. We treat cortices as dissipative systems that self-organize themselves near a critical level of activity that is a non-equilibrium metastable state. Criticality is arguably a key aspect of brains in their rapid adaptation, reconfiguration, high storage capacity, and sensitive response to external stimuli. Self-organized criticality (SOC) became an important concept to describe neural systems. We argue that transitions from one AM pattern to the other require the concept of phase transitions, extending beyond the dynamics described by SOC. We employ random graph theory (RGT) and percolation dynamics as fundamental mathematical approaches to model fluctuations in the cortical tissue. Our results indicate that perceptions are formed through a phase transition from a disorganized (high entropy) to a well-organized (low entropy) state, which explains the swiftness of the emergence of the perceptual experience in response to learned stimuli.

## Introduction

It is now commonplace to regard cerebral cortex as an organ maintaining itself in a dynamic state at the edge of criticality (de Arcangelis et al., 2014; Plenz and Niebur, 2014). Criticality in mathematics and physics relates to a point of sudden transition from one state to another. In thermodynamics, the term denotes a point on the phase boundary between solid, liquid and gas phases. Near the critical point, the state of the system changes drastically with the variation of some control parameter, which behavior has been observed in the operation of the cortex (Freeman, 2008; Fraiman and Chialvo, 2012; Freeman et al., 2012). Metastability is a related fundamental behavior employed in characterizing brain dynamics and cognition (Bressler and Kelso, 2001; Freeman and Holmes, 2005; Tognoli and Kelso, 2014). Metastability indicates a continuous interplay between phase synchrony and phase scattering in a system with many interacting components (van Straaten and Stam, 2013; Zalesky et al., 2014; Freeman, 2015).

How does the cortex maintain a critical state? Nuclear physicists use the concept of criticality to denote the threshold, at which nuclear fission reaction is maintained. The critical state of the fission chain reaction is achieved by a delicate balance between the material composition of the reactor and its geometrical properties. The criticality condition is expressed as the identity of geometrical curvature (buckling) and material curvature. Critical processes in nuclear reactors are designed in a way to satisfy strictly linear operational regimes, in order to guarantee stability of the underlying coupled reactor dynamic process (Upadhyaya et al., 1980; Kozma, 1985; March-Leuba and Rey, 1993). In brains, however, nonlinear feedback effects are of primary importance in sustaining complex cortical dynamics (Kozma and Freeman, 2001; Tagliazucchi and Chialvo, 2012). Our answer to the question on the origin of sustained critical state in brains is that mutual excitation between populations of cortical neurons maintains criticality, in combination with the refractory period that prevents exponential grow, thus stabilizes the dynamics (Freeman, 1975, 2004a).

In the past decade, neuroscientists successfully employed the concept of self-organized criticality (SOC) to neural processes (Beggs, 2008; Friston et al., 2012; Fingelkurts et al., 2013; Palva et al., 2013; Plenz and Niebur, 2014). These and many other studies point to scale-free dynamics in the cortex resembling cascades of sand piles during metastable states (Bak, 1996; Jensen, 1998; Petermann et al., 2009). SOC, however, cannot describe the existence of robust critical regions with sustained metastable dynamics, neither the rapid transitions from one metastable state to the other (Tognoli and Kelso, 2014). Bonachela et al. (2010) describe brains as “pseudo-critical” and suggest that we should “… look for more elaborate (adaptive/evolutionary) explanations, beyond simple self-organization.” Reinforcement learning (RL) is crucial in producing rapid transitions from one metastable state to the other (Freeman, 1979). RL sensitizes the cortex selectively and creates spatially extended Hebbian cell assemblies (HCAs). Once HCAs are formed, they respond collectively to conditional stimuli. Stimulating any part of the assembly triggers a rapid increase in synaptic gain, leading to the explosive increase in the activity, until the activation density reaches saturation (Freeman, 2015). HCAs manifest emergent neural packets facilitating the understanding of perceptual experiences (Yufik and Friston, 2016).

Synchronized bursts of neural activity have been observed and analyzed extensively in the literature. This includes the description of spike bursts in interacting excitatory-inhibitory neural populations (see, e.g., Hindmarsh and Rose, 1984; Izhikevich, 2000; Coombes and Bressloff, 2005; Srinivasan et al., 2013). Mathematical models based on chaos theory have been proved to be useful to describe these bursts patterns (Hansel and Sompolinsky, 1992; Tsuda, 2001; Kozma, 2003). Recent breakthroughs include the comprehensive description of sharp wave ripples representing episodic memory effects (Buzsáki, 2015) and systematic analysis of spike bursts (Werbos and Davis, 2016). Our work addresses experimental and theoretical findings of transient synchronization in mesoscopic neural populations and their interpretation based on the concept of phase transitions in random graph theory (RGT) and statistical physics.

Since the early 2000s, phase transition in RGT has been employed as a useful mathematical concept to model the dynamics of the cortical tissue (Kozma et al., 2001). The random graph description of the cortex, called “*neuropercolation*,” implements a hierarchy of cortical models (Kozma et al., 2005). Non-local interactions between neural populations via long axonal projections are crucial in describing cortical dynamics. There are extensive studies to model small-world effects (Watts and Strogatz, 1998) in structural and functional brain networks tuned to criticality (Bullmore and Sporns, 2009, 2012; Turova, 2012; Haimovici et al., 2013; Sporns, 2013; Alagapan et al., 2016). The level of system noise, the ratio of non-local connections corresponding to long axons, and the strength of inhibitory effects are key variables that allow controlling the transitions between opposite phases (Kozma and Puljic, 2015). In the absence of non-local connections, diffusion-like effects dominate the spatio-temporal dynamics, which fall short of producing the required rapid cortical transitions. With the help of non-local connections, we were able to generate and maintain phase transitions exhibiting rapid transitions between synchronized and desynchronized phases (Puljic and Kozma, 2008, 2010; Kozma and Puljic, 2015).

Phase transitions between disordered and ordered neural states provide key insights to understand and interpret the observed cortical space-time neurodynamics. Disordered states are characterized by random dispersion of active and inactive sites, while the emergence of metastable amplitude modulation (AM) patterns signify more ordered states. In the disorganized phase, the individual microscopic neurons are loosely coupled, which facilitates them processing sensory information individually. In the organized phase, the neurons are strongly coupled into populations producing metastable macroscopic AM patterns (Freeman, 2014). Transitions from one AM pattern to the other produce a sequence of metastable cortical states, which can be viewed as neural correlates of cognitive activity in the framework of the cinematic theory of cognition (Freeman, 2006, 2007; Kozma and Freeman, 2016). The cinematic theory of cognition is related to the concept of perception occurring in discrete epochs (Crick and Koch, 2003), and to the model of pulsating consciousness manifested via neuronal activity packages (Yufik, 2013).

This essay summarizes our decades-long experimental and theoretical studies supporting the concept of the cinematic theory of cognition. We review the theory of criticality in the cerebral cortex based on self-organized dynamics of neural populations, manifested in the form of sequential phase transitions between metastable AM patterns. In our interpretation, phase transitions are responsible for the rapid responses to sensory stimuli observed in cognitive processing and for the emergence of our perceptual experiences according to the cinematic theory of cognition.

## Constructing the Self-Organized Perception Cycle

### Metastable AM Patterns Manifest the Organized Phase of Cortical Dynamics

From the variety of the available brain monitoring techniques, here we focus on recordings EEG and ECoG potentials. Intracranial experiments with electrode arrays over the cortex have been conducted in various laboratories, providing a window on the electrophysiological processes underlying brain functions (Freeman, 1975; Skarda and Freeman, 1987; Canolty et al., 2010; Panagiotides et al., 2011; Buzsáki et al., 2012). A state-of-art overview of brain imaging using EEG and ECoG monitoring techniques is given by Freeman and Quian-Quiroga (2013), including single trial experiments, high-density arrays, and spatio-temporal spectral analysis. More traditional Fourier analysis is often supplemented by Hilbert transform, which is especially beneficial in the characterization of rapidly changing, metastable activity patterns.

**Figure 1:**
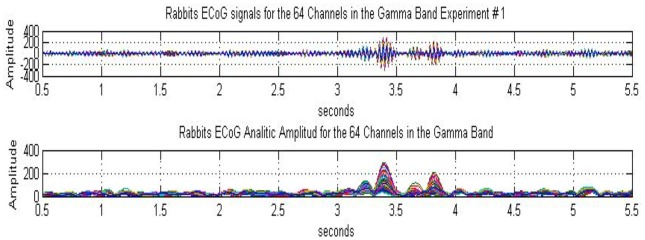
**Rabbit ECoG data measured over the visual cortex using an 8 × 8 array of electrodes.** The duration of the experiment is 6 s, with a visual stimulus (light flash) presented to the animal at *t* = 3 s; the signals were filtered over the gamma band (30–36 Hz). The subplots show 64 curves corresponding to the ECoG signals (top) and the analytic signals (bottom), respectively. The analytic signals have been calculated using Hilbert transform, from Davis et al. (2013).

We illustrate the experimental results concerning the presence of highly organized metastable AM patterns and their intermittent collapse to a disorganized state using the example of rabbits, conducted in the Freeman neurophysiology laboratory at UC Berkeley (Freeman and Barrie, 2000). Rabbits were implanted with intracranial electrode arrays over their sensory cortices and trained using the well established, RL paradigm. In the experiment displayed in Figure 1, an ECoG array of 8 × 8 electrodes is fixed over the visual cortex of the rabbit. The measurement is 6 s long with a visual stimulus presented to the animal at time instant *t* = 3 s; thus there is a 3 s pre-stimulus and a 3 s post-stimulus period. Figure 1, upper plot, shows the 64 ECoG traces filtered in the gamma band 30–36 Hz (Davis et al., 2013). There is a base level of background activity during the 3 s expectancy state without stimulus. During the ~1 s interval following the stimulus several gamma bursts appear. Finally, after about 1 s following the stimulus (at time instants >4 s), the activity returns to the background state. The novelty of the results lies in the development of quantitative measures to characterize the sequence of metastable states, using various pragmatic information indices (Freeman, 2004a; Davis et al., 2013).

Using Hilbert transform for each of the 64 ECoG signals, complex valued analytic signals are obtained with amplitude and phase components. The analytic amplitude represents the power of the ECoG signal, while the phase can be used to monitor synchronization effects. In Figure 1, lower plot, the amplitudes of the 64 analytic signals are shown. In the pre-stimulus period, the amplitudes fluctuate at a low level, indicating a sustained, disorganized background activity. There are several beats during the ~1 s period following the stimulus, which demonstrate intermittent bursts of power in the gamma band. These bursts signify the emergence of metastable AM patterns (for details, see Freeman, 1975, 2004a, 2014).

The existence of an AM pattern indicates that the cortical dynamics is constrained to a narrow attractor basin in response to a given stimulus. This is a highly structured (organized) state with significant coordination between the 64 ECoG channels. In spite of the individual differences between the ECoG channels, they have significant commonality in their behaviors; namely, they rise, reach a maximum, and decrease in synchrony. This means that the AM pattern is largely time-invariant during the 100–200 ms of its existence, although its overall intensity varies in time. The relevance of AM patterns in defining the cognitive state of the animal has been demonstrated by using AM patterns as classification tools to discriminate between stimuli (Freeman, 1979; Kozma and Freeman, 2001). The AM patterns provide us with an observation window to monitor the cognitive process using ECoG/EEG techniques. When the input is removed, the cortical dynamics is released from its constrained state, the AM pattern disappears, and the cortex returns to the disorganized, background state.

The AM patterns do not represent the input stimuli in any practical sense; rather they correspond to the meaning of the input. They continuously change during the life of the animal through a learning process, as a result of past experiences, present state and future goals of the subject. If a new stimulus does not match a previously learnt experience, the response of the cortex is a rapidly decaying oscillation. If the stimulus is presented again and again to the animal, the connections between excitatory neurons are strengthened in a process called Hebbian learning. As the result, the response decays less and less, which ultimately leads to sustained narrow-band oscillations due to the formation of a HCAs. The emergence of narrow-band oscillations is crucial for the efficient memory readout based on metastable AM patterns. The role of Hebbian reinforcement of connections between co-activated neurons has been demonstrated in large neuron populations, including the hippocampus, sensorimotor and speech areas (Buzsáki, 2005; Pulvermüller and Fadiga, 2010; Lopes-dos-Santos et al., 2013). In the computational domain, Hebbian RL has been implemented in various neural network models (see, e.g., Amit, 1995; Wennekers and Palm, 2009).

**Figure 2:**
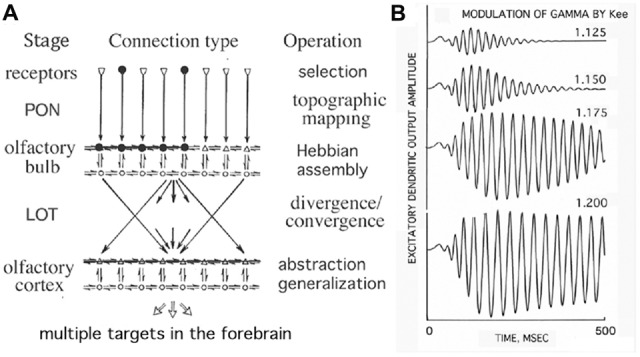
**Illustration of the topographic mapping that characterizes the olfactory input pathway. (A)** Input is transmitted via the primary olfactory nerve (PON) to the olfactory bulb having excitatory (upper) and inhibitory (lower) layers, where the Hebbian assembly is shown (black dots). The Hebbian assembly is ignited with the stimulation of any of its subsets and leads to a phase transition from broad-band background activity to narrow-band oscillations, which are projected to the olfactory cortex through the lateral olfactory tract (LOT). **(B)** The increased strengths of mutual excitatory connections (Kee) in the Hebbian assembly strongly enhance gamma oscillations in response to learnt stimuli (Freeman, 1979).

The example of the olfactory system with convergent-divergent connections is illustrated in Figure 2 (Freeman, 1979). Input is transmitted via the primary olfactory nerve (PON) to the olfactory bulb, where the HCA is shown by black dots. By stimulating any subset of the HCA, the whole HCA is activated and produces narrow-band oscillations, thus exhibits the key property of generalization over the category of the sensory stimulus. Activations from the bulb are projected to the olfactory cortex through the lateral olfactory tract (LOT). The increased strengths of mutual excitatory connections (Kee) in the Hebbian assembly strongly enhance gamma oscillations in response to learned stimuli (Baird et al., 1991; Kozma and Freeman, 2001). In the context of the present work it is to be emphasized that he formation of HCAs and their rapid activation in response to learned stimuli are important conditions of cortical phase transitions (Freeman, 2015).

### Background Activity and “Null Spikes”

The low overall magnitudes of the ECoG and analytic signals in Figure 1 before the stimulus onset (*t* < 3 s) indicate that the background activity is a state of relatively low energy as compared to the high-energy burst of the AM patterns. Moreover, the energy of the background oscillations is distributed over a wide range of frequencies as opposed to the narrow-band (gamma) oscillations contributing the formation of AM patterns. In fact, the background conforms to power-law dynamics with a power exponent ranging between −2 and −4 (Freeman and Zhai, 2009). It is generated by mutual excitation among populations of cortical excitatory neurons, which activity places great demand on bodily metabolism even in brains at rest, sometimes referred to as “dark energy” (Raichle, 2006).

The background activity is characterized by weak correlation and strong desynchronization between individual channels. The overall low background activity level may briefly drop to near zero for some channels, which phenomenon is called *“null spike”* (Freeman, 2008; Kozma and Freeman, 2008). During null spikes, the analytic phase of the background exhibits sudden changes, jumps, discontinuities; the channels have significant dispersion in their analytic phases. If the background is described as a disordered phase compared to the ordered phase with metastable AM patterns, then the null spikes clearly represent extreme disorder, which we characterize as singularity. The singularity is embedded in the background activity. At the singularity, we observe that the analytic amplitude diminishes and the analytic phase dispersion increases explosively. The very low power of the null spike means that the interactions between neural populations are suppressed. This provides favorable conditions for inputs to have a significant impact on the behavior of neural populations, especially through igniting relevant Hebbian assemblies, which facilitate a consequent rapid propagation of activities.

Null spikes are interpreted as the sites of nucleation initiating a phase transition, following the analogy of crystallization or condensation. For example, when a liquid is converted to a solid phase, the solidification starts as a specific point on the surface, and expands from that point rapidly as the liquid to solid phase transition progresses. Similarly, condensation of steam into the liquid phase starts at a point on the surface; the incipient drop grows from that location by expanding the boundary between the liquid and vapor phases. Following these examples, the initiation of null spike on the cortex may signify the start of the phase transition in the brain dynamics from disorganized phase to organized phase. In brains, the organized phase appears in the form of an emergent AM pattern with increasing power at the frequency of the carrier wave (gamma power).

The synchronized pattern emerges at the wake of a phase gradient rapidly propagating over the surface of the cortex. This phase gradient has the form of a cone and it is called *“phase cone”* (Freeman, 2004b). Note that there are many phase cones that appear and disappear all the time, however, those phase cones are mostly small (microscopic), and do not grow to the macroscopic size characteristic of a phase transition. Only when the drop of the analytic power coincides with the presence of a suitable stimulus, can we observe the rapid growth of a phase cone to sizes covering large cortical areas. The location of the apex of the cone varies randomly from each burst to the next and has no relation to the stimulus. The conic apex is in itself a singularity, and there is some preliminary evidence that its location may correspond to the location of the preceding null spike (Freeman, 2015).

### The Collapse of AM Patterns

AM patterns represent highly organized states of the cortex, which ultimately dissolve through gradual erosion under continual bombardment by sensory stimuli. The collapse of AM patterns can be viewed as a phase transitions from a synchronized to a disorganized state. In physics, such a conversion is described as evaporation of a liquid, or melting of a solid substance. This phase transition requires energy transferred to the system.

AM patterns are synchronized bursts of the activities of large masses of neurons, which emerge through phase transitions initiated by null spikes and exists for a fraction of a second (theta rates). There is a characteristic frequency of the burst in the gamma band due to the interaction of excitatory and inhibitory populations, but there is a marked distribution of frequencies of the myriads of individual feedback loops that contribute to the formation of the AM pattern (Kozma and Freeman, 2008). It is inevitable that variations in these frequencies produce oscillations that become less and less synchronized, thus the collective order of the neural populations decreases. As a result, the overall power of the oscillations diminishes and the AM patterns collapse (Freeman, 2014).

The elimination of the AM pattern drives the dynamics back to the background level, which will produce another AM pattern and the whole cycle starts again. The presence of the continual cycle of the emergence and destruction of metastable AM patterns is an important property of cortical dynamics, which is a lifelong process. In the next section, this cycle is discussed in the context of the cinematic theory of cognition, while energy considerations are described afterwards.

## Cinematic Model of Perception as a Sequence of Phase Transitions

ECoG measurements with intermittent transitions between synchronized and desynchronized brain states are interpreted in the framework of the cinematic theory of cognition (Freeman, 2007; Kozma and Freeman, 2016). Accordingly, neocortex processes information in frames like a cinema. Metastable AM patterns manifest the “*frames*,” and the phase transitions provide the *“shutter”* from one frame to the next. Moving from one metastable pattern to the other corresponds to successive images in a movie, which we interpret using the synergetic approach to information processing (Haken, 1983). Haken proposed that state transitions are essential for information transfer between hierarchical levels, by which a collection of particles create an order parameter and in circular causality enslaves the activity of the particles. Cortical AM patterns are the manifestations of the enslavement of individual neural oscillations by collective EEG dynamics (Freeman, 2007).

**Figure 3:**
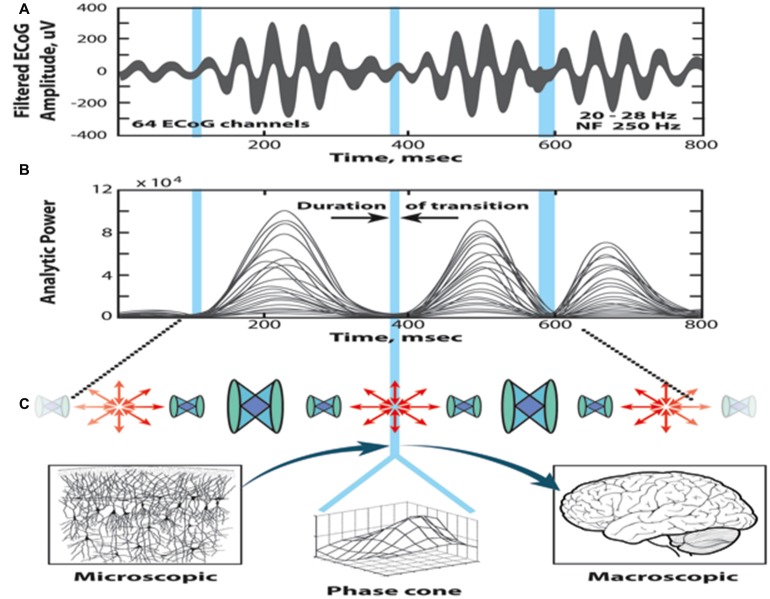
**Illustration of the self-organized perception cycle based on the cinematic theory of cognition. (A)** Superimposed band-pass filtered ECoG signals. **(B)** The 64 analytic amplitudes show beats with high amplitudes, interrupted with periods of reduced power, marked by blue bars (null spikes). The high amplitudes between the blue bars correspond to metastable amplitude modulation (AM) patterns carrying the cognitive content (frames). The null spikes are singularities localized in space and time, with high dispersion of the phases (shutter). **(C)** Following the singularity, large phase cones emerge, which manifest transition from microscopic disorder to macroscopic order (illustration by Chris Gralapp), from Kozma and Freeman (2016).

Figure 3 illustrates the sequential processing in the cinematic model of cognition; the top two diagrams show the superimposed 64 ECoG signals (pass band: 20–28 Hz) and the corresponding curves of the analytic power, respectively. The time evolution displays a sequence of beats having relatively high power, separated by periods with diminishing analytic power (marked by blue vertical bars). The duration of a beat is about 100–200 ms, and a metastable AM pattern is sustained during this period. The blue bars correspond to brief time periods of transition from one beat to the other. During the transition, the AM patterns collapse to a singularity (null spike), when the synchrony disappears and the phase relationships exhibit high dispersion.

The cinematic theory employs two main components of cortical dynamics that occur sequentially, namely, the movie frame and the shutter.

- The frames are defined by the metastable AM patterns, which describe a phase with synchronous activity and macroscopic order. The metastable AM patterns represent a transmission mode of operation, i.e., they convey the knowledge contained in the meaning of the stimulus that gave rise to AM patterns. At the ordered phase, the cortex ignores the impact of the irrelevant input stimuli, until the AM pattern finally erodes and leads to the disorganized phase (shutter).
- The shutter is brief (~20 ms) and it corresponds to the collapse of order due to the desynchronization of the neural activity. This is the receiving phase of the perception cycle, when the analytic power drops near zero and the dynamics becomes susceptible to input stimuli. Once a relevant stimulus is selected, it activates a HCA and induces rapid growth of a large phase cone, which extends over distant cortical areas.

The cinematic theory describes two types of phase transitions, one with the emergence of order from disorder in the form of AM patterns, and the other is the collapse of order manifested in the dissolution of the AM patterns.

- Transition from disorder to order: AM patterns emerge rapidly following the initiation by a null spike under the influence of a relevant stimulus. The large cones are initiated and maintained by corresponding HCAs. These large-scale phase cones enslave the cortical dynamics and lead to the emergence of order in the form of AM patterns. Without activating a HCA, the incipient phase cones cannot grow to macroscopic level, rather they remain localized, and the impact of the input stimuli rapidly fades away.
- Transitions from order to disorder: the degradation of the AM patterns is gradual, under the constant impact of input stimuli. At first, AM patterns are highly synchronized and resist to perturbations in the form of the emergence and collapse of small phase cones during the metastable state. Ultimately, however, the synchrony erodes, the power of the population activity decreases, and the dynamics returns to the disorganized background phase.

The existence of metastable AM patterns and their ultimate collapse can be interpreted in the context of SOC. There are incipient, smaller phase cones during the metastable AM patterns (Freeman, 2004b), which resemble avalanches of various sizes that maintain the state of SOC (Bak, 1996; Jensen, 1998; Beggs and Plenz, 2003). The power law distribution of avalanche sizes suggests that the neural tissue is in the dynamic state of criticality. These incipient phase cones manifest the dissipation of energy in weak bursts. Such incipient cones may manifest the SOC metastable state, however, they are different from the large-scale phase cones emerging during the phase transitions. SOC cannot describe the sequence of transient patterns observed in the perception cycle and described here in the context of the cinematic theory of cognition. Neuropercolation is a suitable mathematical tool to describe cortical phase transitions, as summarized next (Kozma and Puljic, 2015).

## Discussion on Graph Theory Interpretation of Cortical Phase Transitions

**Figure 4:**
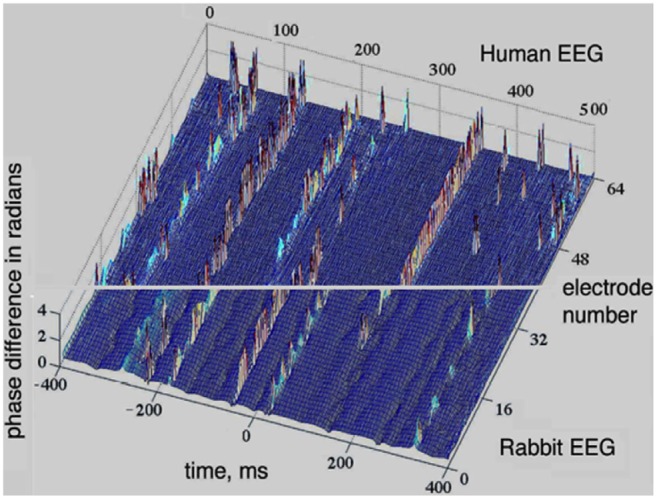
**Synchronization-desynchronization effects seen in EEG measurements with humans (lower part) and ECoG with rabbits (upper part); there are extended periods with low phase differences across space (blue color), interrupted by short periods with large phase differences (variable colors).** The window of the 8 × 8 array was 5.6 × 5.6 mm for the rabbit data (upper half), while a 1 × 64 curvilinear array (189 mm long) was used over the scalp of normal human volunteers (lower half; Freeman, 2004b).

The perception cycle is a sequence of transitions between synchronized and desynchronized states. EEG and ECoG measurements provide a window of observation into this cycle by monitoring synchronization properties of the AM patterns. A prominent example of synchronization-desynchronization transitions in the cortex is depicted in Figure 4, where the analytical phase difference is shown in the vertical axis, against time and space (*x* and *y* axes). Uniformly distributed phase differences indicate synchrony across the array, while highly variable phase differences mark the presence of desynchronization. The upper segment of Figure 4 is based on the 8 × 8 array of electrodes with rabbits, while the lower segment is based on intracranial measurements of the EEG of human volunteers using a linear array of 64 electrodes (Freeman, 2004b). One can see extended periods of global synchrony indicated by dominant blue colors, i.e., uniformly low values of phase differences. The periods of synchrony are interrupted by brief desynchronization events shown by a range of colors due to the large spread of the phase differences.

A family of hierarchical models of cortical dynamics has been developed originally for the olfactory system (Freeman, 1979), which is called now Freeman K (Katchalsky) sets. Freeman K sets have been applied as a general neural network model to describe chaotic dynamic memories using encoding of external data in a sequence of spatial oscillatory patterns, mimicking cortical AM patterns. The original mathematical formulation of the model was based on a set of second-order ordinary differential equations (ODEs) with distributed parameters (for an overview, see Kozma and Freeman, 2001). Freeman K sets have been used in the past decades for pattern recognition, time series prediction, autonomous navigation and control, and clustering in cyber-security domains (Harter and Kozma, 2005; Kozma et al., 2007; Freeman and Kozma, 2010; Rosa and Piazentin, 2016).

An alternative implementation of Freeman K sets uses RGT instead of ODEs and it is called “neuropercolation” (Kozma et al., 2001, 2005; Kozma, 2007). Neuropercolation is based on a mathematical approach combining cellular automata on lattices and random graphs. Neuropercolation considers the interconnected network of neural populations as large-scale random graphs, which exhibit phase transitions near some well-defined critical states. Neuropercolation includes sparse rewiring of connections creating small-world effects (Watts and Strogatz, 1998), as well as the interaction of excitatory and inhibitory populations (Puljic and Kozma, 2008). It has clear advantages as compared to ODEs in characterizing rapid transients and phase transitions, due to the inherent flexibility of the graph theory framework (Kozma and Puljic, 2013, 2015; Janson et al., 2016).

**Figure 5:**
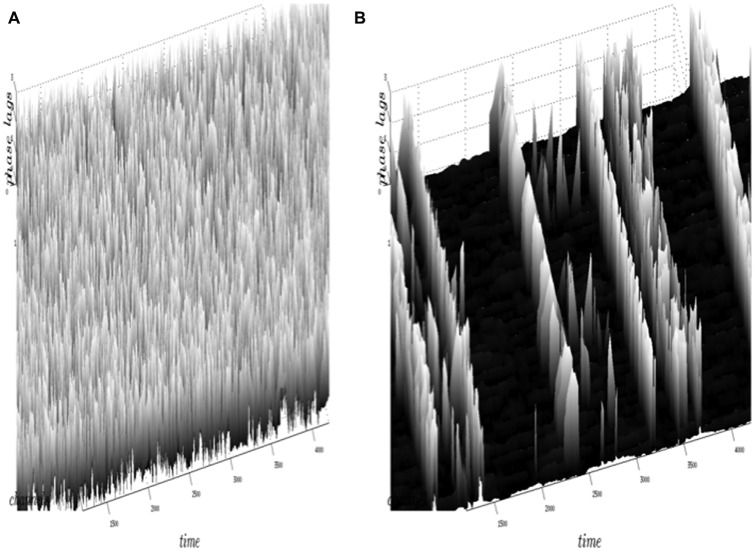
**Illustration of the results obtained by the neuropercolation model of Freeman K sets with excitatory and inhibitory neural populations.** Phase lags (vertical axis) are depicted for individual channels across time (*x* axis) and space (*y* axis). In the model we use the noise level (p) as a control parameter, which allows tuning the system to criticality. The supercritical state **(A)** has highly variable phase differences without synchrony, corresponding to *p* = 0.13. Criticality is obtained in plot **(B)** with probability value *p* = 0.15, which drives the system to spontaneous, intermittent synchronization across the array (Kozma and Puljic, 2013).

Figure 5 illustrates the results obtained by the neuropercolation model using Freeman K sets with excitatory and inhibitory populations. Figure 5A shows the supercritical state with highly variable phase differences (no synchrony), while Figure 5B is an example of the near critical state with intermittent synchronization-desynchronization transitions. The criticality of the system is controlled by the overall noise level (p); *p* = 0.13 belongs to a supercritical state (no synchrony), while *p* = 0.15 results in critical state with synchronization-desynchronization transitions; from Kozma and Puljic (2013). Note that the calculated synchronization-desynchronization transitions across space and time resemble the dynamics observed in measurements with ECoG/EEG arrays. This result supports the hypothesis that the transitions between organized and disorganized phases in the cortex may be the consequence of the cortex residing in a metastable state near criticality.

## Conclusions

Brains constitute only 2% of the human body but they use disproportionately high amount of energy (over 20%), which shows that creating intelligence requires a large amount of metabolic energy. Therefore, energy considerations are very important to understand the nature of biological intelligence in our brains, as well as in attempting to create artificial intelligence in machines.

The cortical energy cycle is summarized as follows, starting from a disordered background state of high entropy and low analytic amplitude. Upon the activation of a HCA by a meaningful stimulus, the synchronized activity of neural populations rapidly propagates across the cortex and creates highly structured AM patterns with low entropy states oscillating in a narrow frequency band (gamma). The formation of AM patterns can be viewed as a condensation process that leads to the dissipation of excess energy in the form of heat that is carried away in the blood stream.

The AM pattern is maintained for some time in a metastable dynamic state that seems to conform to SOC. Synchronized activity of extended neural populations is clearly documented through low phase dispersion between ECoG/EEG channels. Some disturbances in the analytic phase of the cortical tissue appear in the form of small-scale phase cones, which disappear soon after they are formed, obeying the rules of self-similar dynamics of sand piles. The energy released during the formation of the AM pattern is replenished through the metabolism, thus the oxygen debt is repaid (Freeman et al., 2012; Freeman, 2014).

The synchrony represented in the AM pattern is under constant threat by the bombardment of input stimuli and it leads to a degradation of the structure, which can be viewed as an evaporation process. Consequently, the neurons uncouple their dynamics as they are released from the binding represented by the structure. Ultimately, the AM pattern disintegrates, the overall level of firing activity decreases, and the analytic amplitude diminishes. The system returns to a chaotic background state and the cycle is completed (for a detailed description of the cycle, see Kozma and Freeman, 2016).

EEG/ECoG techniques provide insight on the perception cycle in the cortex. Synchronization-desynchronization transitions can be measured by noninvasive scalp EEG (Ruiz et al., 2010; Panagiotides et al., 2011), which allows monitoring the cognitive activity of normal subjects during routine daily activities (Freeman and Quian-Quiroga, 2013). This creates the opportunity to develop various brain-computer interfaces to improve the quality of life of the healthy human population and people with disabilities.

## Author Contributions

All authors listed, have made substantial, direct and intellectual contribution to the work, as displayed in the publication.

## Conflict of Interest Statement

The authors declare that the research was conducted in the absence of any commercial or financial relationships that could be construed as a potential conflict of interest. The reviewer PJW declared a past co-authorship with one of the authors RK to the handling Editor, who ensured that the process met the standards of a fair and objective review.
